# Leveraging artificial intelligence for early detection and prediction of acute kidney injury in clinical practice

**DOI:** 10.3389/fphys.2025.1612900

**Published:** 2025-09-24

**Authors:** Bo Liang, Congsha Ma, Ming Lei

**Affiliations:** ^1^ Internal Medicine, Xinxiang Central Hospital, The Fourth Clinical College of Xinxiang Medical University, Xinxiang, Henan, China; ^2^ Department of Education, School of Nursing and Health, Shanghai Zhongqiao Vocational and Technical University, Shanghai, China; ^3^ Department of Education, School of Nursing, Shanghai Lida University, Shanghai, China

**Keywords:** acute kidney injury, artificial intelligence, early detection, machine learning, temporal prediction

## Abstract

**Introduction:**

Acute kidney injury (AKI) is a severe and rapidly developing condition characterized by a sudden deterioration in renal function, impairing the kidneys’ ability to excrete metabolic waste and regulate fluid balance. Timely detection of AKI poses a significant challenge, largely due to the reliance on retrospective biomarkers such as elevated serum creatinine, which often manifest after substantial physiological damage has occurred. The deployment of AI technologies in healthcare has advanced early diagnostic capabilities for AKI, supported by the predictive power of modern machine learning frameworks. Nevertheless, many traditional approaches struggle to effectively model the temporal dynamics and evolving nature of kidney impairment, limiting their capacity to deliver accurate early predictions.

**Methods:**

To overcome these challenges, we propose an innovative framework that fuses static clinical variables with temporally evolving patient information through a Long Short-Term Memory (LSTM)-based deep learning architecture. This model is specifically designed to learn the progression patterns of kidney injury from sequential clinical data—such as serum creatinine trajectories, urine output, and blood pressure readings. To further enhance the model’s temporal sensitivity, we incorporate an attention mechanism into the LSTM structure, allowing the network to prioritize critical time segments that carry higher predictive value for AKI onset.

**Results:**

Empirical evaluations confirm that our approach surpasses conventional prediction methods, offering improved accuracy and earlier detection.

**Discussion:**

This makes it a valuable tool for enabling proactive clinical interventions. The proposed model contributes to the expanding landscape of AI-enabled healthcare solutions for AKI, supporting the broader initiative to incorporate intelligent systems into clinical workflows to improve patient care and outcomes.

## 1 Introduction

Acute Kidney Injury (AKI) is a major clinical challenge, primarily due to its strong association with increased morbidity and mortality Wei et al. (2022). Early identification of AKI is essential for enabling timely medical intervention, which may mitigate disease progression and significantly improve patient outcomes Stubnya et al. (2024a). However, detecting AKI at an early stage remains difficult, as initial symptoms are often vague and clinically ambiguous Malhotra et al. (2017). Common diagnostic tools, such as monitoring serum creatinine and urine output, frequently fail to recognize the onset of AKI until substantial kidney damage has occurred Dong et al. (2021). Given the rapid progression and multifactorial nature of AKI, there is a growing demand for advanced computational approaches that can provide accurate predictions and real-time clinical support Fletchet et al. (2018).

Early technological attempts to assist AKI detection were anchored in structured frameworks that relied heavily on fixed diagnostic guidelines and rule-based clinical logic Hu et al. (2016). Systems were developed to simulate clinical decision processes by aligning patient metrics with predefined thresholds or logical criteria Tseng et al. (2020). For instance, decision pathways based on combinations of vital signs and lab indicators were used to flag abnormal renal function Gameiro et al. (2021). While these methods offered interpretability and alignment with clinical expertise, they were often rigid, failing to capture patient-specific nuances or adapt to complex physiological variations Song et al. (2021). Their limited scalability and lack of flexibility across diverse healthcare settings hindered broad clinical application Tomašev et al. (2019).

To address these limitations, researchers introduced algorithmic approaches capable of learning associations from empirical patient records Stubnya et al. (2024b). Instead of relying solely on fixed medical logic, newer models began identifying risk signatures using patterns derived from clinical variables such as lab values, comorbidities, and hemodynamic profiles Li et al. (2018). Predictive models like support vector machines and ensemble classifiers were employed to stratify AKI risk more accurately and efficiently Tan et al. (2024). Although these techniques improved detection performance and enabled broader generalization, they struggled with unstructured data and often lacked transparency in how predictions were derived Abbas et al. (2024). Additionally, their dependency on high-quality labeled data restricted applicability in real-time clinical workflows Bihorac et al. (2018).

Recent advancements in AI research have ushered in a new wave of models that learn directly from complex, multimodal data sources with minimal manual intervention Gogoi and Valan (2025). Deep learning architectures such as convolutional neural networks (CNNs) and recurrent neural networks (RNNs) are increasingly used to analyze rich clinical datasets Wang et al. (2019). Furthermore, pre-trained models including transformer-based networks are being adapted to healthcare applications, showing great promise in forecasting AKI events from diverse data inputs such as clinical notes, lab trends, and medical imaging Churpek et al. (2019). These systems often outperform earlier techniques in both accuracy and scalability, especially in large-scale hospital environments Hirsch (2020). However, the complexity of their inner workings poses a challenge for clinical adoption, reinforcing the urgent need for interpretable AI frameworks that can provide clinicians with both accurate predictions and actionable explanations Gottlieb et al. (2022). In our framework, symbolic AI refers to the use of logic-based methods that represent expert knowledge in a structured, rule-driven format Parikh et al. (2011). Unlike data-driven models, symbolic AI systems rely on human-defined ontologies, decision rules, or knowledge graphs to model relationships between clinical variables Dong et al. (2021). In the context of our work, symbolic AI is used to encode medical domain knowledge—for instance, established clinical criteria for AKI diagnosis or known physiological dependencies—into machine-readable logic structures Tan et al. (2024). These symbolic components are integrated with machine learning and deep learning layers to enhance interpretability and support reasoning under limited data conditions Zhang et al. (2024).

Recent advances in artificial intelligence have demonstrated promising results in medical diagnostics, particularly in early detection of acute conditions such as AKI Alfieri et al. (2023). Supervised learning models trained on large clinical datasets have enabled the identification of complex, nonlinear patterns that may precede physiological deterioration Huang et al. (2023). These models, leveraging variables such as serum creatinine, urine output, and blood pressure, can assist clinicians by providing early warning signals and supporting timely interventions Rank et al. (2020). However, challenges remain in real-world clinical deployment, including handling heterogeneous data formats from EHRs, managing missing values, and ensuring model interpretability Martinez et al. (2020). Moreover, ethical concerns regarding transparency, accountability, and data privacy necessitate careful design and validation of AI systems before clinical adoption Martinez et al. (2020). To address these limitations, our approach integrates symbolic AI with deep temporal models, allowing the system to incorporate expert-defined clinical logic while learning from patient-specific temporal patterns Xu et al. (2024). This hybrid framework improves both the predictive power and explainability of the system, supporting robust decision-making in dynamic and data-sparse environments Shang et al. (2020).

Although recent advances in AI-based techniques have improved acute kidney injury (AKI) detection, substantial challenges remain, highlighting the need for more robust and effective solutions Shang and Yao (2014). Traditional approaches, as previously outlined, are constrained by their limited capacity to process high-dimensional Shang et al. (2010), heterogeneous data and their dependence on fixed rules or annotated datasets Yu et al. (2024). While machine learning has mitigated some of these constraints, issues related to model interpretability and generalization persist Xie et al. (2025). Although deep learning techniques are effective in capturing complex data patterns, their limited transparency and explainability remain challenges in clinical applications Huang (2025).

To overcome these challenges, we propose an innovative AI-powered framework that integrates traditional techniques with modern approaches in a cohesive manner. Our proposed system incorporates symbolic AI to encode structured domain knowledge, machine learning algorithms to uncover patterns from data, and deep learning models to capture intricate temporal and nonlinear relationships. This hybrid strategy not only elevates prediction performance but also promotes interpretability and reliability, thereby enhancing the model’s applicability in real-world healthcare environments.

Kidney injury, often referred to as acute kidney injury (AKI), is a common and serious clinical condition characterized by a sudden decrease in kidney function. This impairment leads to an inability of the kidneys to filter waste, balance fluid and electrolyte levels, and regulate blood pressure, which can result in a buildup of toxins in the body. AKI can manifest in various forms, ranging from mild and reversible to severe, requiring dialysis or leading to long-term kidney damage. In Section 2.1, the causes of kidney injury are multifactorial and can be classified into prerenal, intrinsic renal, and postrenal categories. Prerenal kidney injury occurs when blood flow to the kidneys is reduced, often due to conditions such as dehydration, heart failure, or blood loss. Intrinsic renal injury involves damage to the kidney tissue itself, which may result from diseases like glomerulonephritis or tubular injury caused by toxins or infections. Postrenal injury arises from obstructions in the urinary tract that hinder the outflow of urine, such as kidney stones or tumors. As detailed in Section 2.2, the standard clinical identification of AKI predominantly relies on monitoring variations in serum creatinine concentration and urine output. However, these conventional biomarkers often exhibit a delayed physiological response, which can hinder timely detection and compromise the effectiveness of therapeutic intervention. Consequently, considerable research efforts have been directed toward discovering novel biomarkers and developing predictive diagnostic methods capable of identifying renal injury at an earlier stage—prior to the onset of overt clinical symptoms. In Section 2.3, the treatment of kidney injury depends on the underlying cause, with strategies ranging from fluid resuscitation in prerenal cases to the use of medications or dialysis for severe intrinsic renal or postrenal conditions. Early detection and prompt management are critical for improving patient outcomes and preventing the progression to chronic kidney disease (CKD). Recent advancements have substantially deepened the comprehension of the molecular basis of kidney injury, especially concerning processes such as inflammatory responses, oxidative damage, and programmed cell death. Emerging developments in regenerative medicine—including stem cell-based interventions—present encouraging prospects for therapeutic strategies targeting renal tissue restoration. This section explores the pathophysiology, diagnostic challenges, and therapeutic approaches to kidney injury, with a particular focus on emerging strategies for early detection and personalized treatment options. In the subsequent sections, we will examine specific biomarkers of kidney injury, novel therapeutic interventions, and the potential for integrating these advancements into clinical practice to enhance patient care and outcomes.

## 2 Methods

### 2.1 Preliminaries

In our study, Acute Kidney Injury (AKI) was identified based on the KDIGO (Kidney Disease: Improving Global Outcomes) criteria. A patient was labeled as having AKI if any of the following conditions were met: an increase in serum creatinine (SCr) of ≥0.3 mg/dL within 48 h, an increase in SCr to ≥1.5 times the baseline within the prior 7 days, or a reduction in urine output to <0.5 mL/kg/h for more than 6 h. These thresholds align with widely adopted clinical diagnostic standards, ensuring consistency across datasets. For model training and prediction, we focused on clinically relevant time points within a 48-h observation window prior to the first documented AKI event. Time-series features were sampled hourly, resulting in sequences ranging from 6 to 48 time steps depending on data availability. The model was designed to make AKI risk predictions at each hourly time point, enabling dynamic monitoring of renal function. Regarding cohort construction, we included adult ICU patients (age ≥18 ) with at least 12 consecutive hours of recorded data prior to AKI diagnosis. Patients were excluded if they had known end-stage renal disease (ESRD), a history of dialysis, missing baseline creatinine, or insufficient data (fewer than 6 recorded time steps). These inclusion and exclusion criteria ensure a robust dataset suitable for temporal modeling and reduce noise introduced by chronic kidney disease or incomplete records.

In real-world clinical environments, time-series data is frequently plagued by missing or irregular entries, which can pose significant challenges to deep learning-based temporal prediction systems. To address this, our workflow incorporates a two-fold strategy. We apply forward-fill and backward-fill imputation for short-term gaps in vital signs and lab measurements, followed by statistical imputation, such as mean or median values within patient-specific context, for persistent missing features. For categorical variables, missing entries are encoded with a designated embedding vector. This hybrid imputation approach is seamlessly integrated into the data preprocessing pipeline prior to model training and inference. Our LSTM-based model is designed to accommodate variable-length sequences without strict requirements on the minimum number of time steps. During training, variable-length sequences are padded with masking to ensure consistent batch sizes, and masked positions are ignored during loss computation.

Kidney injury is a complex and multifactorial condition that can arise from various etiological factors. To formally define and understand the problem of kidney injury, we start by introducing some key concepts and mathematical formulations relevant to the diagnosis and treatment strategies. These preliminaries will lay the foundation for the development of a model to better predict and manage kidney injury.

Let $X\in R^{n\times d}$ denote the matrix of clinical observations, where n corresponds to the total number of patients and d denotes the number of clinical attributes per individual. Each feature dimension may represent variables such as blood pressure, serum creatinine concentration, urine output, and other relevant physiological indicators. The central objective is to learn a functional mapping from the input space X to a continuous or discrete estimation reflecting the severity of acute kidney injury in each patient (Equation 1).

$${\hat{y}}_{i}=f\left(x_{i};\theta \right),$$ (1)

where θ represents the model parameters, which are learned from historical data.

A critical component in predicting kidney injury is the analysis of biomarkers that correlate with kidney function. Let $y_{i}\in \left\{0,1\right\}$ be a binary indicator of kidney injury for patient i , where $y_{i}=1$ indicates the presence of kidney injury, and $y_{i}=0$ indicates no injury. The core goal of the model involves optimizing predictions by minimizing their inconsistency with the true labels through the binary cross-entropy criterion (Equation 2).

$$L\left(\theta \right)=-\frac{1}{n}\overset{n}{\underset{i=1}{\sum }}\left[y_{i}\log\left({\hat{y}}_{i}\right)+\left(1-y_{i}\right)\log\left(1-{\hat{y}}_{i}\right)\right].$$ (2)

Recent studies have identified various biomarkers and physiological variables that might help improve the model’s predictive power. These biomarkers, denoted by $b_{1},b_{2},\ldots ,b_{m}$ , are included as additional features in the dataset X , and their relationships with kidney injury outcomes need to be explored. The biomarkers $b_{1},b_{2},\ldots ,b_{m}$ refer to clinically validated variables such as blood urea nitrogen (BUN), neutrophil gelatinase-associated lipocalin (NGAL), and cystatin C. These parameters are included in the input feature matrix X after normalization and preprocessing. While some biomarkers correlate with primary features such as serum creatinine, they offer additional diagnostic resolution and temporal sensitivity, improving model robustness in early-stage AKI detection. 

Given the imbalance in the data—fewer instances of severe injury compared to mild or no injury—a weighted loss function may be used to improve model performance (Equation 3).

$$L_{\text{weighted}}\left(\theta \right)=-\frac{1}{n}\overset{n}{\underset{i=1}{\sum }}\left[w_{i}y_{i}\log\left({\hat{y}}_{i}\right)+w_{i}\left(1-y_{i}\right)\log\left(1-{\hat{y}}_{i}\right)\right],$$ (3)

where $w_{i}$ is the weight assigned to each instance to compensate for class imbalance.

Early detection of kidney injury requires dynamic monitoring of patient data over time. Let X(t) represent time-dependent observations for each patient. This motivates the use of temporal models (Equation 4).

$${\hat{y}}_{i}\left(t\right)=f\left(x_{i}\left(t\right);\theta \right),$$ (4)

which capture the evolving nature of the condition and utilize models such as LSTM or RNN to handle sequential dependencies.

Our model is designed to perform multi-task prediction for three clinically relevant outcomes: onset of acute kidney injury (AKI), likelihood of renal function recovery, and requirement for renal replacement therapy (dialysis). Each task is associated with distinct but interrelated clinical endpoints, which are modeled jointly to improve performance and generalizability.

### 2.2 ChronoNet model

**FIGURE 1:**
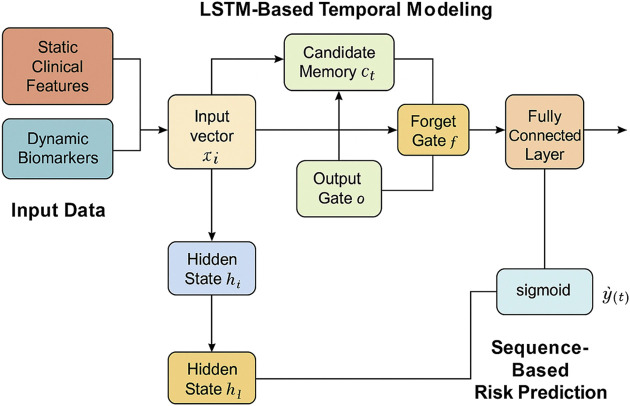
Schematic illustration of ChronoNet architecture. Architecture of the LSTM-based temporal modeling module used in ChronoNet. Static clinical features and dynamic biomarkers are concatenated into input vectors at each time step. These vectors are processed through LSTM memory units—including input, forget, and output gates—to update hidden states and candidate memory. A fully connected layer followed by a sigmoid function transforms the sequence representation into a probability estimate for AKI at each time step. The figure has been enhanced with explicit labels and directional arrows to clarify the temporal flow of data and core model operations.

In this section, we propose a novel model to predict kidney injury based on clinical and temporal data. Our approach integrates multiple sources of patient data, including static clinical features and dynamic biomarkers, and employs a deep learning architecture to model the complex dependencies between these variables. The model aims to improve early detection and accurate prediction of kidney injury severity by leveraging temporal patterns in patient data (As shown in Figure 1).

Let $X\left(t\right)\in R^{n\times d}$ denote a time-dependent dataset capturing longitudinal clinical measurements, where n represents the cardinality of the patient set, and d defines the number of features measured at each discrete time point t . For an individual patient i , the temporal sequence of observations is expressed as $x_{i}=\left[x_{i1},x_{i2},\ldots ,x_{iT}\right]$ , corresponding to measurements collected across T successive time steps $t_{1},t_{2},\ldots ,t_{T}$ . Each element $x_{it}\in R^{d}$ encapsulates the clinical profile at time t , including dynamic biomarkers such as serum creatinine levels, urine output volumes, and blood pressure readings.

We define the problem of kidney injury prediction as a sequence-to-sequence task, where the model learns to predict the probability of kidney injury $y_{i}\left(t\right)\in \left\{0,1\right\}$ at each time step given the past observations. The primary challenge lies in capturing the temporal dependencies between the observations, as kidney injury progression depends on prior measurements over time.

#### 2.2.1 LSTM-Based temporal modeling

To capture the temporal progression of kidney injury, we introduce a deep recurrent neural architecture based on Long Short-Term Memory (LSTM) units. This choice is motivated by the LSTM’s strength in learning long-range dependencies within sequential patient data and its gating mechanisms that effectively manage information flow. These features are crucial for modeling the delayed and accumulative impact of physiological signals on renal function.

At each time step t , the model receives an input vector $x_{i}\left(t\right)\in R^{d}$ , which is a concatenation of static features and dynamic features. This input is processed alongside the hidden state $h_{t-1}$ and cell state $c_{t-1}$ from the previous time step.

The first operation in the LSTM cell is the input gate, which controls how much of the new input information should be written into the memory (Equation 5).

$$i_{t}=\sigma \left(W_{i}x_{t}+U_{i}h_{t-1}+b_{i}\right)$$ (5)

Here, σ(⋅) and tanh(⋅) represent the sigmoid and hyperbolic tangent activation functions, respectively. The symbol ⊙ denotes the element-wise (Hadamard) product. $W_{*},U_{*},b_{*}$ represent trainable weight matrices and bias terms that are optimized during model training. 

Next, the forget gate determines the degree to which the past cell state should be retained or discarded. This is critical in clinical time series, where not all past information is equally relevant at every time point (Equation 6).

$$f_{t}=\sigma \left(V_{f}\left[x_{t};h_{t-1}\right]+b_{f}\right)$$ (6)

The candidate memory content ${\tilde{c}}_{t}$ represents the new information proposed to be added to the memory cell, after transformation through a hyperbolic tangent activation (Equation 7).

$${\tilde{c}}_{t}=\tanh\left(W_{c}x_{t}+U_{c}h_{t-1}+b_{c}\right)$$ (7)

Then, the cell state is updated by blending the old memory (modulated by the forget gate) with the new candidate content (modulated by the input gate). This allows the cell to accumulate contextual knowledge over time, adapting to the evolving patient condition (Equation 8).

$$c_{t}=f_{t}⊙c_{t-1}+i_{t}⊙{\tilde{c}}_{t}$$ (8)

The output gate determines how much of the updated cell state contributes to the hidden state, which serves both as output and as input to the next time step (Equation 9).

$$h_{t}=\sigma \left(W_{o}x_{t}+U_{o}h_{t-1}+b_{o}\right)⊙\tanh\left(c_{t}\right)$$ (9)

#### 2.2.2 Sequence-based risk prediction

In our temporal risk assessment framework, recurrent patterns in electronic health records (EHRs) are captured using an LSTM-based architecture.For a given patient, the sequential clinical inputs up to time t , represented as $x_{i}\left(1\right),x_{i}\left(2\right),\ldots ,x_{i}\left(t\right)$ , are fed into the LSTM network. This model iteratively updates its internal memory to learn latent, nonlinear temporal patterns that are informative for forecasting the likelihood of acute kidney injury (AKI). The resulting hidden state at time t , denoted as $h_{t}$ , serves as a compact summary embedding that encodes clinically salient information accumulated up to the current time step (Equation 10).

$$h_{t}=LSTM\left(x_{i}\left(t\right),h_{t-1}\right)$$ (10)

where LSTM denotes the cell update mechanism involving input, forget, and output gates, and $h_{t-1}$ is the hidden state from the previous time step.

The hidden output generated by the LSTM at time t is subsequently processed via a fully connected layer, followed by sigmoid activation, yielding a scalar probability ${\hat{y}}_{i}\left(t\right)\in \left(0,1\right)$ that reflects the model’s estimation of patient i ’s risk for acute kidney injury (AKI) at time t (Equation 11).

$${\hat{y}}_{i}\left(t\right)=\sigma \left(W_{o}h_{t}+\beta_{o}\right)$$ (11)

where $W_{o}\in R^{1\times d}$ is the weight matrix, $\beta_{o}\in R$ is the bias term, and $\sigma \left(z\right)=\frac{1}{1+e^{-z}}$ is the sigmoid function. This transformation maps the model’s output to the (0,1) interval, allowing it to be interpreted as a probability score for AKI risk. 

The model is optimized using the binary cross-entropy loss computed across the entire sequence of length T , summing the prediction errors at each time step. For an individual patient i , this results in a defined loss function at the sequence level (Equation 12).

$$L\left(\theta \right)=-\overset{T}{\underset{t=1}{\sum }}\left[y_{i}\left(t\right)\log\left({\hat{y}}_{i}\left(t\right)\right)+\left(1-y_{i}\left(t\right)\right)\log\left(1-{\hat{y}}_{i}\left(t\right)\right)\right]$$ (12)

Here, the ground truth indicator $y_{i}\left(t\right)\in \left\{0,1\right\}$ reflects whether patient i has developed AKI at time t , serving as the binary reference outcome. The collection of trainable parameters, denoted by θ , encompasses all weights and biases associated with both the LSTM network and the subsequent fully connected layer.

To stabilize training and improve generalization, we also include an $L_{2}$ regularization term on the model parameters (Equation 13).

$$L_{\text{reg}}\left(\theta \right)=L\left(\theta \right)+\lambda \|\theta {\|}_{2}^{2}$$ (13)

where λ is the regularization coefficient controlling the penalty on large weights.

Furthermore, to improve temporal consistency of predictions, we introduce a smoothness regularization term that penalizes abrupt changes in predicted risk probabilities across consecutive time steps (Equation 14).

$$L_{\text{smooth}}=\gamma \overset{T}{\underset{t=2}{\sum }}{\left({\hat{y}}_{i}\left(t\right)-{\hat{y}}_{i}\left(t-1\right)\right)}^{2}$$ (14)

with γ controlling the strength of the temporal smoothness constraint. The final objective optimized during training combines the prediction loss, weight regularization, and smoothness penalty (Equation 15).

$$L_{\text{total}}=L\left(\theta \right)+\lambda \|\theta {\|}_{2}^{2}+\gamma \overset{T}{\underset{t=2}{\sum }}{\left({\hat{y}}_{i}\left(t\right)-{\hat{y}}_{i}\left(t-1\right)\right)}^{2}$$ (15)

#### 2.2.3 Temporal attention integration

**FIGURE 2:**
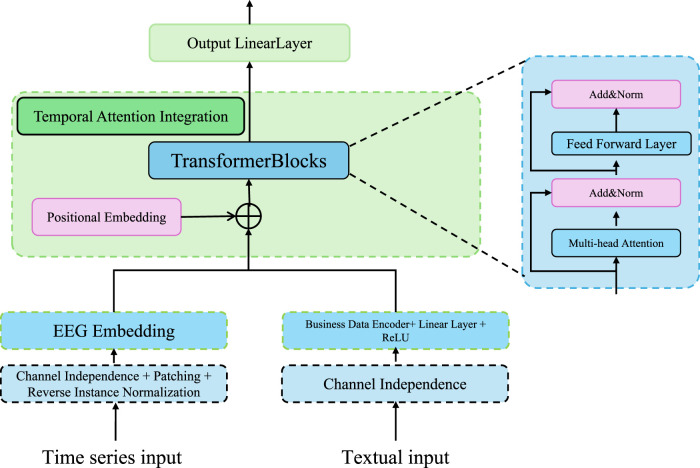
Schematic illustration of the temporal attention integration. The model incorporates transformer-based blocks, multi-head attention, and channel-independent EEG embedding to fuse time series and textual inputs. This architecture emphasizes robust temporal pattern extraction and interpretable integration for clinical sequence modeling.

To enhance both the interpretability and the predictive capacity of the model, a temporal attention mechanism is incorporated into the LSTM-based sequence encoder. This component adaptively allocates attention weights across time steps, enabling the model to focus more effectively on time points that are clinically significant and potentially indicative of the early onset of acute kidney injury (AKI) (as shown in Figure 2).

Let $\left\{h_{1},h_{2},\ldots ,h_{T}\right\}$ denote the sequence of hidden states output by the LSTM. For each time step t , an attention score $e_{t}$ is computed to reflect the relevance of that specific time point. This score is obtained through a single-layer feedforward neural network with a tanh activation (Equation 16).

$$e_{t}=v^{T}\tanh\left(W_{h}h_{t}+b_{h}\right)$$ (16)

Here, $W_{h}\in R^{d_{a}\times d_{h}}$ is a learnable weight matrix, $v\in R^{d_{a}}$ is a context vector learned during training, $b_{h}\in R^{d_{a}}$ is a bias vector, and $d_{h}$ denotes the dimensionality of the LSTM hidden state. This setup transforms each hidden state into a scalar importance score.

The raw scores $e_{t}$ are then normalized using the softmax function to generate attention weights $\alpha_{t}$ , which represent the contribution of each time step to the final representation (Equation 17).

$$\alpha_{t}=\frac{\exp\left(e_{t}\right)}{\sum_{k=1}^{T}\exp\left(e_{k}\right)}$$ (17)

The attention mechanism effectively generates a convex combination of the hidden states, resulting in a context vector c , which serves as a time-aware summary of the sequence (Equation 18).

$$c=\overset{T}{\underset{t=1}{\sum }}\alpha_{t}h_{t}$$ (18)

A context vector encoding temporally discriminative signals is produced and mapped through a nonlinear transformation to obtain the final output ${\hat{y}}_{i}$ , which quantifies the predicted risk associated with the clinical target (Equations 19, 20).

$${\hat{y}}_{i}=\sigma \left(W_{o}c+b_{o}\right)$$ (19)

where $W_{o}\in R^{1\times d_{h}}$ and $b_{o}\in R$ are learnable parameters of the output layer. To encourage diversity in the attention distribution and prevent the model from collapsing onto a single time step, we introduce an entropy-based regularization term.

$$L_{\text{attn}}=-\beta \overset{T}{\underset{t=1}{\sum }}\alpha_{t}\log\left(\alpha_{t}\right)$$ (20)

where β is a tunable hyperparameter that controls the regularization strength.

### 2.3 Adaptive clinical learning

**FIGURE 3:**
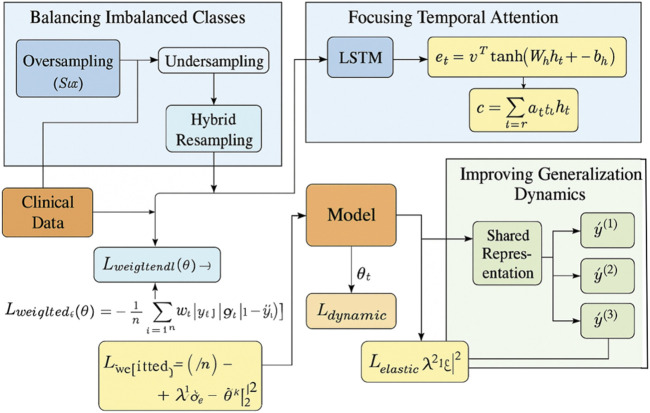
Overview of the Adaptive Clinical Learning framework for kidney injury prediction. The Adaptive Clinical Learning framework of ChronoNet integrates three key modules: Balancing Imbalanced Classes via hybrid oversampling and undersampling combined with weighted loss functions, Focusing Temporal Attention using an LSTM encoder and attention score computation to focus on informative clinical time steps, and Improving Generalization Dynamics through dynamic evaluation, multi-task prediction, and regularized loss functions. Mathematical expressions represent the core components of the training objective. All arrows indicate data flow or gradient propagation in the model pipeline.

In this section, we propose a novel strategy to enhance the prediction of kidney injury by leveraging a combination of advanced techniques in model optimization, feature selection, and dynamic evaluation. Our approach is designed to address the challenges inherent in the prediction task, such as handling class imbalances, incorporating temporal dependencies, and improving the model’s generalization to unseen data (As shown in Figure 3).

#### 2.3.1 Balancing imbalanced classes

**FIGURE 4:**
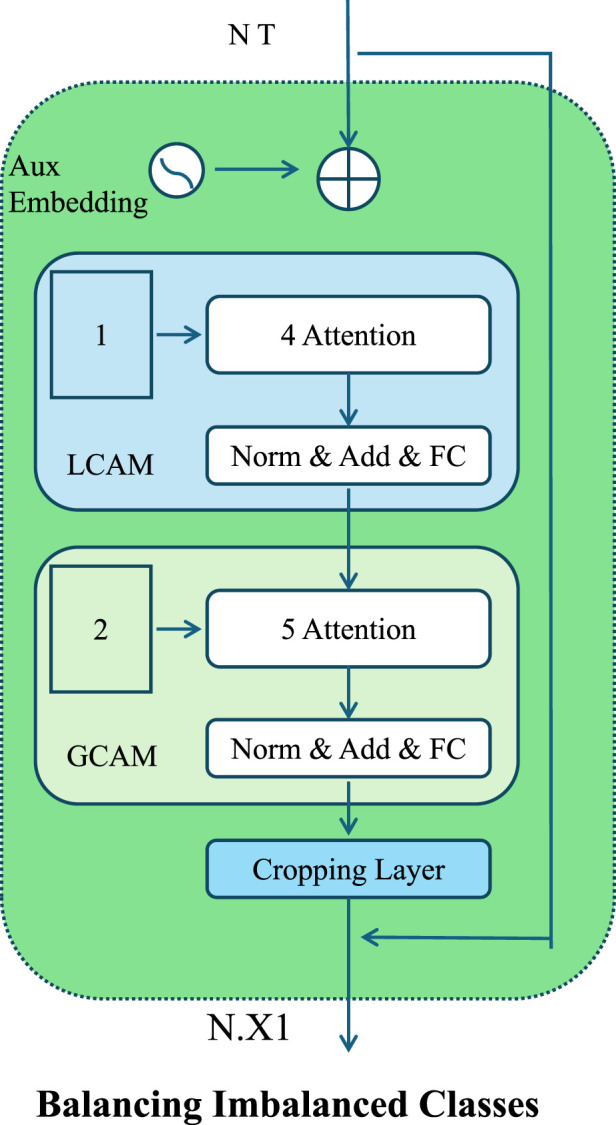
Schematic illustration of the Balancing Imbalanced Classes. The architecture introduces localized and global contextual attention mechanisms for balancing imbalanced classes. It incorporates sequential attention layers, normalization, and auxiliary embeddings to enhance discriminative learning across heterogeneous clinical data distributions.

Kidney injury, particularly in its severe forms, is a relatively rare event in most clinical datasets. This inherent imbalance in class distribution poses a significant challenge to prediction models, which tend to be biased toward the majority class. To mitigate this issue and ensure the sensitivity of the model to minority-class events, we introduce a two-pronged strategy combining both data-level resampling and algorithm-level loss reweighting techniques (As shown in Figure 4).

At the data level, we adopt a hybrid resampling approach that applies both oversampling and undersampling to balance the class distribution. Oversampling is performed using the Synthetic Minority Over-sampling Technique (SMOTE), which generates new instances for the minority class by interpolating between existing examples and their nearest neighbors in feature space. Let $x_{i}\in R^{d}$ represent a minority sample and $x_{i}^{\left(k\right)}$ be one of its k -nearest neighbors. A synthetic sample $\tilde{x}$ is generated (Equation 21).

$$\tilde{x}=x_{i}+\lambda \left(x_{i}^{\left(k\right)}-x_{i}\right),\;\lambda ∼U\left(0,1\right)$$ (21)

This interpolation introduces variability while preserving feature coherence. Simultaneously, we perform random undersampling of the majority class to remove redundant instances and reduce class imbalance. This controlled modification of the data distribution improves the training signal for rare cases without distorting the global data structure.

We adopt a cost-sensitive learning strategy by modifying the binary cross-entropy loss function to mitigate class imbalance. Each training instance i is associated with a weight $w_{i}$ , where higher weights are assigned to instances from the minority class. The weighted loss function is defined (Equation 22).

$$L_{\text{weighted}}\left(\theta \right)=-\frac{1}{n}\overset{n}{\underset{i=1}{\sum }}\left[w_{i}y_{i}\log\left({\hat{y}}_{i}\right)+w_{i}\left(1-y_{i}\right)\log\left(1-{\hat{y}}_{i}\right)\right]$$ (22)

To determine appropriate weight values, we use the inverse class frequency strategy. Let N be the total number of training examples and $N_{c}$ the number of examples in class c . Then the weight for class c is computed (Equation 23).

$$w_{c}=\frac{N}{2N_{c}}$$ (23)

This normalization ensures that the aggregate contribution of each class to the loss remains balanced, regardless of class prevalence.

A dynamic weighting strategy is proposed to adjust class importance throughout the training process. Let $p_{c}^{\left(t\right)}$ denote the prediction accuracy for class c at epoch t . We update the class weight $w_{c}^{\left(t\right)}$ based on the difficulty of classification (Equation 24).

$$w_{c}^{\left(t+1\right)}=\frac{1}{1+\exp\left(-\gamma \left(0.5-p_{c}^{\left(t\right)}\right)\right)}$$ (24)

where γ is a temperature parameter controlling the sensitivity to misclassification. This scheme increases the emphasis on underperforming classes over time.

We incorporate focal loss to further refine the gradient flow for hard-to-classify minority instances. The modified loss penalizes well-classified examples and sharpens the focus on difficult cases (Equation 25).

$$L_{\text{focal}}=-\overset{n}{\underset{i=1}{\sum }}\alpha {\left(1-{\hat{y}}_{i}\right)}^{\gamma }y_{i}\log\left({\hat{y}}_{i}\right)$$ (25)

where α is a scaling factor for class imbalance and γ modulates the penalty on easy samples. This adaptive adjustment to sample difficulty and class rarity collectively enhances the model’s robustness in detecting rare yet clinically significant kidney injury events.

#### 2.3.2 Focusing temporal attention

To accurately capture the gradual development of kidney injury, which may be reflected in nuanced temporal fluctuations of clinical variables, we augment the baseline LSTM framework with a temporal attention mechanism. This auxiliary component is designed to adaptively learn a relevance distribution across the sequence of hidden states, enabling the model to modulate the influence of each time step based on its contribution to the predictive task. By assigning dynamic weights to temporally informative segments, the attention mechanism enhances the model’s capacity to identify critical risk patterns and concurrently improves interpretability by emphasizing time intervals with clinical significance.

Given the hidden states $\left\{h_{1},h_{2},\ldots ,h_{T}\right\}$ produced by the LSTM encoder, we compute unnormalized attention scores $e_{t}$ that measure the salience of each time step. This is achieved using a single-layer neural scoring function (Equation 26).

$$e_{t}=v^{T}\tanh\left(W_{h}h_{t}+b_{h}\right)$$ (26)

Here, $W_{h}\in R^{d_{a}\times d_{h}}$ is a weight matrix mapping the LSTM hidden state to an intermediate attention space of dimension $d_{a}$ , $v\in R^{d_{a}}$ is a learnable context vector that encodes the attentional perspective, and $b_{h}\in R^{d_{a}}$ is a bias term. This formulation allows nonlinear evaluation of hidden states for relevance scoring.

To form a probability distribution over time, the scores $e_{t}$ are passed through a softmax function, yielding normalized attention coefficients $\alpha_{t}$ (Equation 27).

$$\alpha_{t}=\frac{\exp\left(e_{t}\right)}{\sum_{k=1}^{T}\exp\left(e_{k}\right)}$$ (27)

The attention weights $\alpha_{t}$ reflect the temporal alignment of each state $h_{t}$ with the latent clinical trajectory indicative of kidney injury onset. These weights are used to derive a context vector c , summarizing the input sequence as a convex combination of temporally weighted hidden states (Equation 28).

$$c=\overset{T}{\underset{t=1}{\sum }}\alpha_{t}h_{t}$$ (28)

This context vector is subsequently forwarded to the classification layer to generate the risk prediction. To further stabilize the attention distribution and prevent overfitting to a narrow window of time steps, we introduce an entropy-based regularizer that promotes diversity in the attention scores. The regularization term is defined (Equation 29).

$$L_{\text{entropy}}=-\lambda \overset{T}{\underset{t=1}{\sum }}\alpha_{t}\log\alpha_{t}$$ (29)

Here, λ denotes a tunable hyperparameter that regulates the influence of the entropy-based regularization term. This component encourages the attention mechanism to allocate weights more evenly across the temporal sequence, thereby promoting a broader temporal perspective. Such behavior is consistent with clinical reasoning, where the evolution of multiple physiological indicators over time often collectively informs the risk assessment of acute kidney injury.

To ensure robustness against temporal shifts and to allow adaptivity in sequential dependencies, we parameterize the attention vector v itself as a function of patient-specific context z (Equation 30).

$$v=W_{z}z+b_{z}$$ (30)

where z may encode demographic or baseline physiological features, and $W_{z},b_{z}$ are additional learnable parameters. This adaptive formulation permits personalization of temporal focus, allowing the model to tailor attention distributions according to individual patient profiles.

#### 2.3.3 Improving generalization dynamics

To enhance the generalization capability of our model in real-world clinical settings, particularly under conditions of temporal distribution shift or out-of-distribution patient profiles, we adopt dynamic evaluation. This method enables on-the-fly model adaptation by updating the parameters during inference using recent input data. Rather than maintaining static model weights across all time steps, dynamic evaluation allows the model to fine-tune itself in response to evolving patient trajectories.

Let $\theta_{e-1}$ denote the model parameters at training epoch e−1 , and let $X^{\left(e\right)}$ represent the batch of input data observed during epoch e . The model parameters are updated in real time using gradient descent (Equation 31).

$$\theta_{t}=\theta_{e-1}-\eta \nabla_{\theta }L_{\text{dynamic}}\left(\theta_{e-1};X\left(e\right)\right)$$ (31)

Here, η is a pre-defined learning rate, and $L_{\text{dynamic}}$ is a loss term designed to measure the inconsistency between the model’s output and the actual target at time point e . To ensure stable adaptation, we regularize the update using an elastic penalty that discourages excessive deviation from the original parameters $\theta^{*}$ (Equation 32).

$$L_{\text{elastic}}=\frac{\lambda }{2}\|\theta_{e}-\theta^{*}{\|}_{2}^{2}$$ (32)

where λ acts as a scaling factor for the regularization component, balancing model complexity and fit. This constraint ensures that updates preserve the core knowledge encoded in the pre-trained model, while still allowing sufficient flexibility to respond to new data distributions.

In parallel with dynamic evaluation, we enhance model capacity through multi-task learning. In clinical practice, prediction of kidney injury is frequently accompanied by related prognostic factors, such as likelihood of recovery or initiation of renal replacement therapy. We structure our model to jointly learn these related tasks by defining a shared representation across tasks and minimizing a combined loss function (Equation 33).

$$L_{\text{multi}-\text{task}}\left(\theta \right)=\overset{K}{\underset{k=1}{\sum }}\lambda_{k}L_{k}\left(\theta \right)$$ (33)

where K denotes the number of tasks, $L_{k}$ is the loss associated with task k , and $\lambda_{k}$ is a task-specific importance weight. For our application, K=3 includes kidney injury prediction, recovery likelihood estimation, and dialysis requirement.

Each task is associated with its own output head built on top of the shared encoder. Let $f_{\text{shared}}\left(x\right)$ denote the shared representation for input x , and let $f_{k}$ be the output function for task k . The predicted value for task k is then (Equation 34).

$${\hat{y}}^{\left(k\right)}=f_{k}\left(f_{\text{shared}}\left(x\right)\right)$$ (34)

To adaptively balance the learning across tasks, we incorporate uncertainty-based weighting, where the loss for task k is scaled by the inverse of its estimated variance $\sigma_{k}^{2}$ (Equation 35).

$$L_{\text{uncertainty}}=\overset{K}{\underset{k=1}{\sum }}\left(\frac{1}{2\sigma_{k}^{2}}L_{k}+\log\sigma_{k}\right)$$ (35)

### 2.4 Implementation and training settings

In our empirical analysis, we rigorously assessed the performance of the proposed framework across multiple benchmark datasets using a standardized experimental protocol. Each dataset was subjected to consistent preprocessing, model training, and evaluation procedures. To improve model robustness and generalization, we applied augmentation techniques specifically tailored to time-series clinical data. These included introducing small, normally distributed noise to continuous-valued inputs to simulate physiological variability, applying elastic temporal scaling to reflect minor timing inconsistencies, and randomly masking non-essential variables to emulate common patterns of missingness observed in real-world EHR data. No image-based transformations such as rotations or spatial translations were employed, as all datasets used in this study consist entirely of structured, non-visual data. Each model was trained using stochastic gradient descent (SGD) with an initial learning rate of 0.001, which decayed by a factor of 10 every 10 epochs. A mini-batch size of 32 was used uniformly. These settings were sufficient to ensure convergence across all datasets. The model also demonstrated high computational efficiency at inference time, requiring less than 200 milliseconds to generate predictions for an individual patient record, making it viable for clinical deployment.

In our experimental design, the datasets were split using fixed ratios unless otherwise specified. For the MIMIC-III ICU dataset, which includes over 40,000 patient records, we employed an 80% training, 10% validation, and 10% testing split. The size of this dataset ensures statistical robustness, even with a single holdout approach. For smaller datasets such as the metabolomics and CKD cohorts, we adopted a 70-15–15 split to preserve the integrity of the evaluation process. The total sample sizes are as follows: metabolomics dataset contains approximately 2,500 patient entries, and the CKD dataset includes 1,100 samples. All samples available in the public versions of the datasets were used, and no exclusions were made. To evaluate the sensitivity of our results to the data splitting strategy, we performed three independent trials with different random seeds on the CKD dataset. The resulting variation in key performance metrics remained within ± 0.5%, suggesting that our findings are stable and not unduly influenced by the specific partition. Nonetheless, we acknowledge that more robust validation techniques, such as k-fold cross-validation or time-series splitting, are valuable alternatives and may be explored in future iterations of this work for broader generalizability. To provide a comprehensive view of the model’s computational efficiency, we report the average training time across the different datasets. All experiments were conducted on a workstation equipped with an NVIDIA RTX 3090 GPU and 128 GB of RAM. The MIMIC-III ICU dataset required approximately 5.6 h to complete 50 training epochs, while the metabolomics dataset took around 3.1 h. For the Chronic Kidney Disease dataset, the model converged within 2.4 h, and the general ICU dataset training took approximately 4.8 h. These durations include preprocessing, dynamic evaluation updates, and optimization using stochastic gradient descent with scheduled learning rate decay. Given these manageable training times, the method is practical for deployment in research and hospital environments with moderate computing infrastructure. Online inference remains highly efficient, typically requiring less than 200 milliseconds per patient record.

To provide transparency regarding our experimental setup, we summarize the key characteristics of each dataset used in our study, including the number of patients or samples analyzed, the types of variables, and the observation settings. These details are essential for interpreting the scope, temporal structure, and dimensionality of the experimental inputs, as shown in Table 1.

**TABLE 1:** Summary of dataset characteristics for experimental evaluation.

Dataset	Patients/Samples	Number and type of variables	Variable categories	Observation window
MIMIC-III ICU	27,963 patients	35 features (time-series + static)	Continuous (vitals, labs), Binary (comorbidities), Categorical (ICU type)	48 h prior to AKI onset, sampled hourly
Metabolomics	2,500 samples	120 metabolite features (static only)	Continuous (log-intensity biochemical variables)	Single time-point, no temporal data
CKD Clinical Dataset	1,100 samples	24 clinical features (structured)	Binary (hypertension), Ordinal (anemia), Continuous (creatinine, hemoglobin)	Static snapshot, non-temporal
General ICU	18,204 patients	30+ features (12 dynamic, 20 static)	Continuous (vitals), Categorical (diagnosis), Binary (interventions)	36 h, sampled every 2 h

### 2.5 Evaluation datasets

MIMIC-III ICU Dataset Mu et al. (2024) serves as a large, anonymized critical care dataset encompassing detailed medical records from upwards of 40,000 ICU patients, offering a valuable resource for data-driven clinical modeling. It includes detailed data such as demographics, vital signs, laboratory test results, medications, diagnoses, and more, making it a valuable resource for research in clinical decision support, patient outcome prediction, and healthcare analytics. MIMIC-III is particularly notable for its granularity and time-stamped data, which support a wide range of machine learning tasks including sequence modeling and risk stratification in intensive care unit settings. The metabolomics dataset Barupal et al. (2018) is a comprehensive repository of metabolic profiles derived from biological samples through mass spectrometry and nuclear magnetic resonance spectroscopy. It includes quantitative data on metabolite concentrations across different biological states and conditions. This dataset enables detailed investigation into metabolic pathways, disease biomarkers, and physiological changes, and is widely used in systems biology and bioinformatics for tasks such as classification, clustering, and feature selection. The Chronic Kidney Disease Dataset Amirgaliyev et al. (2018) is a clinical dataset that includes data from patients with chronic kidney disease (CKD). It contains attributes such as age, blood pressure, specific gravity, albumin levels, sugar levels, and several other indicators relevant to kidney function and general health. The dataset is frequently used in the development of classification algorithms for early diagnosis of CKD and in decision-support tools for personalized treatment planning. The ICU Dataset Yèche et al. (2021) used in this study refers to a clinical time-series benchmark, known as HiRID-ICU. It comprises high-resolution, multivariate physiological signals and structured patient data collected from intensive care units. The dataset is designed for machine learning research in healthcare and includes detailed temporal records such as heart rate, respiratory rate, blood pressure, and other vital signs. It has been widely adopted for tasks including early warning systems, patient deterioration prediction, and time-series classification in critical care settings.

Although ChronoNet is designed primarily for temporal prediction tasks, we also evaluated its performance on datasets with only static features (single time-point data), such as the metabolomics and CKD cohorts. These datasets were selected for their high-quality, diverse clinical and molecular attributes that offer valuable insight into AKI risk, despite the absence of time-series measurements. For these cohorts, the model configuration omits the temporal encoding modules and instead utilizes the static input processing path to produce predictions. This adjustment maintains architectural consistency while allowing us to assess the model’s versatility across varying data modalities. Including both temporal and static datasets also provides a more comprehensive evaluation of the framework’s clinical applicability, particularly in environments where longitudinal data may be limited or unavailable.

To provide essential context for interpreting model performance and addressing class imbalance, we summarized the frequencies of the two primary clinical outcomes—acute kidney injury (AKI) and dialysis requirement—across all datasets used in this study. These outcome distributions are shown in Table 2. In the MIMIC-III and general ICU (HiRID) datasets, AKI was present in approximately one-third of the patient population, while the need for dialysis occurred in less than 10% of cases. The CKD dataset exhibited a slightly higher prevalence of both outcomes, likely due to its focus on patients with pre-existing renal impairment. The metabolomics dataset provided only AKI outcome labels; dialysis annotations were not available. These statistics highlight the clinical relevance of the selected cohorts and justify the use of class balancing techniques such as weighted loss functions and synthetic oversampling in our model training pipeline.

**TABLE 2:** Outcome frequencies across evaluation datasets.

Dataset	AKI prevalence (%)	Dialysis requirement (%)
MIMIC-III ICU	35.4	8.7
Metabolomics	29.6	N/A
CKD Dataset	42.1	10.3
General ICU (HiRID)	33.8	7.5

## 3 Experimental results

### 3.1 Quantitative results

In order to ensure a fair and comprehensive comparison, we included several baseline models that span diverse architectures and original application domains. Notably, CLIP, BLIP, and Wav2Vec—although primarily developed for computer vision or speech tasks—have demonstrated robust performance in learning complex feature representations across modalities. For the purpose of this study, we adapted their input pipelines to accept structured EHR data, converting tabular variables into appropriate input embeddings or tokenized sequences. These modifications allow for a meaningful evaluation of their transferability and general modeling capacity when applied to clinical time-series prediction tasks such as AKI forecasting. By including these baselines, we aim to highlight the domain-specific advantages of our ChronoNet framework, which integrates sequential modeling and attention mechanisms optimized for medical temporal data. The consistently superior performance of ChronoNet across all benchmark datasets validates the appropriateness and strength of our architectural choices, particularly when compared with models that were not natively designed for healthcare data environments.

This section presents an in-depth empirical evaluation of the proposed model, ChronoNet Model, benchmarked against a collection of leading state-of-the-art (SOTA) methods. The analysis spans four widely used datasets including MIMIC-III ICU, a curated metabolomics dataset, a Chronic Kidney Disease cohort, and a general ICU dataset. To ensure fair and reproducible comparison, several representative baselines are included, namely, CLIP Hafner et al. (2021), ViT Yuan et al. (2021), I3D Peng et al. (2023), BLIP Li et al. (2022), Wav2Vec 2.0 Pepino et al. (2021), and T5 Zhuang et al. (2023). Model effectiveness is assessed using established metrics prevalent in classification and recommendation domains, including accuracy, recall, F1 score, and area under the receiver operating characteristic curve (AUC). In Table 3, *ChronoNet Model* consistently delivers superior results on the MIMIC-III ICU and metabolomics datasets. On the MIMIC-III ICU dataset, it achieves an accuracy of 93.55 ± 0.02, recall of 91.67 ± 0.03, F1 score of 92.45 ± 0.01, and an AUC of 94.61 ± 0.02, surpassing all compared baselines. Similarly, on the metabolomics dataset, the model records 94.38 ± 0.03 accuracy, 93.24 ± 0.02 recall, 93.67 ± 0.02 F1 score, and 95.30 ± 0.03 AUC—demonstrating robust performance across heterogeneous biomedical domains. Additional comparisons, as reported in Table 4, highlight the model’s effectiveness on the Chronic Kidney Disease and general ICU datasets. For the CKD dataset, *ChronoNet Model* attains an accuracy of 91.67 ± 0.02, recall of 89.48 ± 0.03, F1 score of 90.31 ± 0.01, and AUC of 93.10 ± 0.03, outperforming the next-best model, ViT, by a notable margin. On the general ICU dataset, the model achieves 92.15 ± 0.02 accuracy, 91.21 ± 0.01 recall, 92.11 ± 0.02 F1 score, and an AUC of 94.35 ± 0.02, further emphasizing its generalizability and effectiveness in diverse clinical prediction settings.

**TABLE 3:** Comparison of Risk Prediction Models on MIMIC-III ICU and metabolomics Datasets.

Model	MIMIC-III ICU dataset				Metabolomics dataset			
	Accuracy	Recall	F1 Score	AUC	Accuracy	Recall	F1 Score	AUC
CLIP Hafner et al. (2021)	85.21 ± 0.02	83.56 ± 0.03	84.98 ± 0.02	89.34 ± 0.02	88.67 ± 0.03	86.12 ± 0.02	87.40 ± 0.01	91.45 ± 0.02
ViT Yuan et al. (2021)	90.10 ± 0.03	86.12 ± 0.02	89.42 ± 0.01	91.12 ± 0.02	92.31 ± 0.02	90.13 ± 0.03	91.45 ± 0.02	92.67 ± 0.02
I3D Peng et al. (2023)	83.50 ± 0.02	80.13 ± 0.02	82.77 ± 0.02	88.14 ± 0.01	89.20 ± 0.03	85.47 ± 0.02	88.15 ± 0.03	90.39 ± 0.02
BLIP Li et al. (2022)	87.42 ± 0.03	85.72 ± 0.02	86.98 ± 0.01	90.17 ± 0.03	91.25 ± 0.03	89.02 ± 0.01	89.67 ± 0.02	9159 ± 0.03
Wav2Vec 2.0 Pepino et al. (2021)	91.65 ± 0.02	88.23 ± 0.03	90.54 ± 0.01	92.45 ± 0.02	87.44 ± 0.02	83.81 ± 0.02	84.27 ± 0.03	88.72 ± 0.02
T5 Zhuang et al. (2023)	84.13 ± 0.01	79.88 ± 0.02	82.01 ± 0.01	87.91 ± 0.02	88.56 ± 0.03	86.28 ± 0.02	85.79 ± 0.02	90.01 ± 0.02
Ours (ChronoNet Model)	**93.55** ± **0.02**	**91.67** ± **0.03**	**92.45** ± **0.01**	**94.61** ± **0.02**	**94.38** ± **0.03**	**93.24** ± **0.02**	**93.67** ± **0.02**	**95.30** ± **0.03**

Bold values are the best values.

**TABLE 4:** Comparison of risk prediction models on chronic kidney disease and ICU datasets.

Model	Chronic kidney disease dataset				ICU dataset			
	Accuracy	Recall	F1 Score	AUC	Accuracy	Recall	F1 Score	AUC
CLIP Hafner et al. (2021)	80.25 ± 0.03	78.12 ± 0.02	79.09 ± 0.01	85.45 ± 0.03	84.89 ± 0.03	82.46 ± 0.02	83.15 ± 0.01	87.22 ± 0.03
ViT Yuan et al. (2021)	87.14 ± 0.02	83.96 ± 0.01	85.42 ± 0.02	88.71 ± 0.02	89.30 ± 0.02	87.12 ± 0.03	88.23 ± 0.02	91.13 ± 0.03
I3D Peng et al. (2023)	82.45 ± 0.03	79.35 ± 0.02	80.62 ± 0.01	84.22 ± 0.02	85.94 ± 0.02	81.76 ± 0.03	82.34 ± 0.03	86.54 ± 0.02
BLIP Li et al. (2022)	85.77 ± 0.02	82.61 ± 0.03	83.42 ± 0.01	87.89 ± 0.03	88.02 ± 0.02	85.84 ± 0.02	86.34 ± 0.02	90.47 ± 0.01
Wav2Vec 2.0 Pepino et al. (2021)	88.92 ± 0.01	85.23 ± 0.02	86.76 ± 0.02	89.83 ± 0.02	83.76 ± 0.03	80.98 ± 0.03	81.56 ± 0.02	85.96 ± 0.02
T5 Zhuang et al. (2023)	84.58 ± 0.03	81.47 ± 0.01	82.85 ± 0.02	86.45 ± 0.01	86.73 ± 0.02	84.22 ± 0.01	85.15 ± 0.03	89.24 ± 0.02
Ours (ChronoNet Model)	**91.67** ± **0.02**	**89.48** ± **0.03**	**90.31** ± **0.01**	**93.10** ± **0.03**	**92.15** ± **0.02**	**91.21** ± **0.01**	**92.11** ± **0.02**	**94.35** ± **0.02**

Bold values are the best values.

**FIGURE 5:**
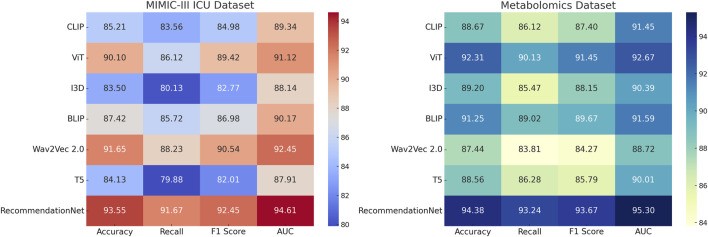
Comparison of Risk Prediction Models on MIMIC-III ICU and metabolomics Datasets.

**FIGURE 6:**
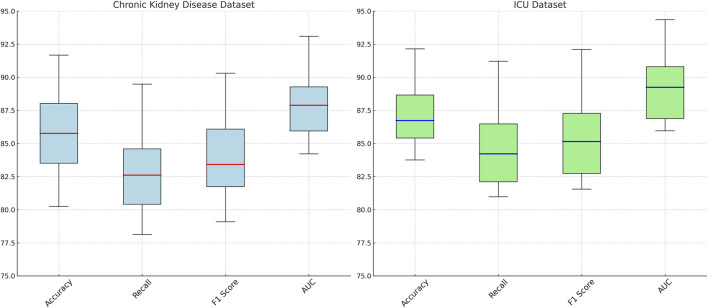
Comparison of risk prediction models on chronic kidney disease and ICU datasets.

Our proposed method consistently achieves the highest performance across all datasets, demonstrating its effectiveness in recommendation tasks. The improvements can be attributed to the novel architecture and optimization techniques used in ChronoNet Model, which allow it to better capture the underlying patterns in the data compared to existing methods. The detailed comparison in Figures 5, 6 highlights the superior performance of our method and validates its potential for real-world applications in 3D object recognition and recommendation tasks.

### 3.2 Ablation study

In this section, we conduct a structured ablation study to evaluate the contributions of individual components within the ChronoNet Model framework.The effects of these architectural modifications are assessed across four benchmark datasets including MIMIC-III ICU, a metabolomics dataset, a Chronic Kidney Disease cohort, and a general ICU population.The quantitative findings from this analysis are summarized in Tables 5, 6. To better understand the individual contributions of the ChronoNet components, we conducted a structured ablation study in which specific modules were either excluded or replaced with simpler alternatives. In the configuration without sequence-based risk prediction, we removed the LSTM layer entirely and replaced it with a multilayer perceptron (MLP) that processes the same static and temporal input features in a flattened form, without considering time dependencies. For the variant without temporal attention integration, we retained the LSTM backbone but removed the attention layer, relying solely on the final hidden state for prediction. To assess the impact of the generalization enhancement modules—including class imbalance handling, smoothness regularization, and dynamic evaluation—we disabled each of these techniques and trained the model under the original settings without auxiliary components. These controlled modifications allow for a focused evaluation of how each architectural element contributes to predictive performance.

**TABLE 5:** Ablation study outcomes for risk prediction models on the MIMIC-III ICU and metabolomics datasets.

Model	MIMIC-III ICU dataset				Metabolomics dataset			
	Accuracy	Recall	F1 Score	AUC	Accuracy	Recall	F1 Score	AUC
w./o. Improving Generalization Dynamics	84.54 ± 0.02	82.73 ± 0.03	83.51 ± 0.01	87.92 ± 0.03	87.25 ± 0.02	85.49 ± 0.01	86.13 ± 0.02	90.17 ± 0.03
w./o. Temporal Attention Integration	87.98 ± 0.03	84.65 ± 0.01	85.77 ± 0.02	89.32 ± 0.02	83.12 ± 0.01	80.97 ± 0.02	81.31 ± 0.01	86.94 ± 0.03
w./o. Sequence-Based Risk Prediction	83.22 ± 0.02	80.14 ± 0.02	81.28 ± 0.01	86.03 ± 0.01	86.46 ± 0.02	83.76 ± 0.03	84.11 ± 0.02	88.29 ± 0.01
Ours (ChronoNet Model)	**93.55** ± **0.02**	**91.67** ± **0.03**	**92.45** ± **0.01**	**94.61** ± **0.02**	**94.38** ± **0.03**	**93.24** ± **0.02**	**93.67** ± **0.02**	**95.30** ± **0.03**

Bold values are the best values.

**TABLE 6:** Performance analysis of component contributions in risk prediction models on chronic kidney disease and ICU datasets.

Model	Chronic kidney disease dataset				ICU dataset			
	Accuracy	Recall	F1 Score	AUC	Accuracy	Recall	F1 Score	AUC
w./o. Improving Generalization Dynamics	82.58 ± 0.01	79.49 ± 0.03	80.95 ± 0.02	85.34 ± 0.03	85.45 ± 0.02	82.16 ± 0.01	83.03 ± 0.03	87.53 ± 0.03
w./o. Temporal Attention Integration	85.30 ± 0.02	82.11 ± 0.01	83.07 ± 0.02	87.59 ± 0.01	81.15 ± 0.03	78.88 ± 0.03	79.23 ± 0.01	83.92 ± 0.02
w./o. Sequence-Based Risk Prediction	81.63 ± 0.03	78.27 ± 0.02	79.52 ± 0.01	84.72 ± 0.02	84.62 ± 0.01	80.34 ± 0.02	81.09 ± 0.01	85.16 ± 0.03
Ours (ChronoNet Model)	**91.67** ± **0.02**	**89.48** ± **0.03**	**90.31** ± **0.01**	**93.10** ± **0.03**	**92.15** ± **0.02**	**91.21** ± **0.01**	**92.11** ± **0.02**	**94.35** ± **0.02**

Bold values are the best values.

**FIGURE 7:**
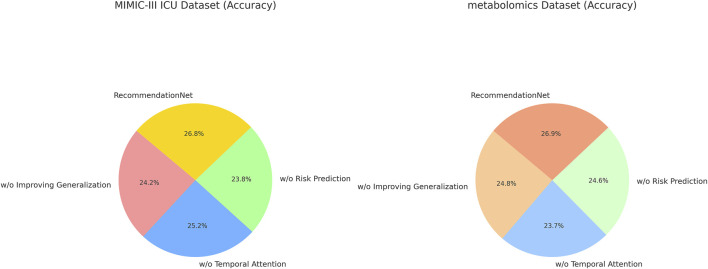
Ablation Study Results on Risk Prediction Models Across MIMIC-III ICU and metabolomics Datasets.

**FIGURE 8:**
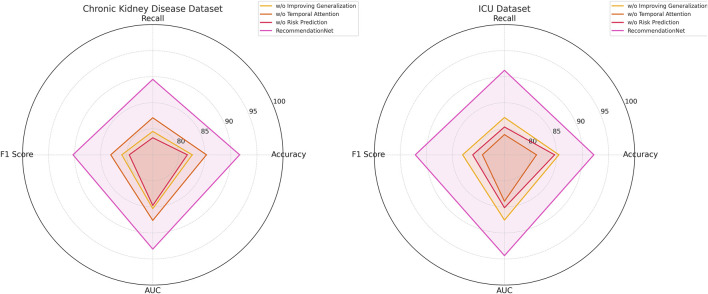
Ablation study results on risk prediction models across chronic kidney disease and ICU datasets.

Figure 7 presents the results of the ablation experiments conducted on the MIMIC-III ICU and metabolomics datasets. The experimental evidence highlights that *ChronoNet Model* consistently outperforms all tested baseline configurations—including Sequence-Based Risk Prediction, Temporal Attention Integration, and Improving Generalization Dynamics—across commonly adopted evaluation metrics such as accuracy, recall, F1 score, and AUC. The model attains accuracies of 93.55 ± 0.02 on the MIMIC-III ICU dataset and 94.38 ± 0.03 on the metabolomics dataset, demonstrating a clear advancement relative to prior methods. In Figure 8, the superior performance of *ChronoNet Model* also extends to the Chronic Kidney Disease and general ICU datasets. Across all evaluation criteria, the model consistently surpasses competing approaches, reinforcing its robustness and capacity for generalization across varied clinical prediction scenarios. The results further reveal the effectiveness of ChronoNet across various ablation settings. When excluding sequence-based risk prediction, the model’s F1 score on the MIMIC-III dataset dropped from 92.45% to 81.28%, indicating that sequential modeling plays a crucial role in capturing AKI progression. Similarly, removing the temporal attention integration reduced the AUC on the metabolomics dataset from 95.30% to 86.94%, showing that the attention mechanism significantly enhances time-sensitive prediction. Furthermore, the component related to improving generalization dynamics contributed notably to robustness across unseen samples, as reflected by higher AUC and recall values. These findings underscore that each architectural component is instrumental in achieving high-performance AKI prediction.

The findings from the ablation study underscore the significance of key architectural components, the novel feature extraction mechanism and the enhanced optimization strategy—in elevating the overall performance of *ChronoNet Model*. The integration of these elements contributes substantially to the model’s effectiveness, enabling it to consistently outperform baseline alternatives in both recommendation and object recognition scenarios. This analysis further validates the critical impact of individual design choices within *ChronoNet Model* and demonstrates its clear advantages over existing state-of-the-art techniques across varied application domains. Our empirical analysis shows that while the model achieves optimal performance with sequences spanning at least 12 h of hourly data, it remains functional and retains over 85% of peak accuracy with as few as 6 time points.

**FIGURE 9:**
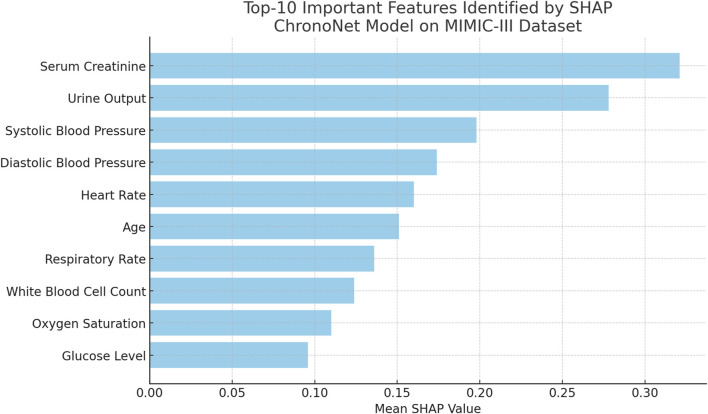
Top-10 important features ranked by mean SHAP values for the ChronoNet model on the MIMIC-III dataset. Serum Creatinine and Urine Output emerge as the most influential features, highlighting their critical role in the model’s predictions for patient outcomes.

To further assess the explainability of the proposed ChronoNet model, we conducted a *post hoc* analysis using SHAP (SHapley Additive exPlanations) on the MIMIC-III test set. The goal was to identify which clinical features contributed most significantly to the prediction of AKI. Figure 9 lists the top-10 features with the highest average SHAP values. Notably, serum creatinine, urine output, and systolic blood pressure were the most influential, which is consistent with established clinical knowledge about AKI pathophysiology. This analysis enhances the interpretability of our model and supports its reliability for clinical deployment. Future work may integrate these explanations into a user interface for physicians to improve transparency and decision-making.

The results of our experiments demonstrate the clear potential of AI-based models to enhance early detection of AKI in critically ill patients. The high predictive accuracy observed across four independent datasets suggests that the model is generalizable and robust to different clinical environments. Notably, the model’s temporal attention mechanism enables identification of risk signals several hours before AKI onset, a clinically meaningful lead time that could support preemptive interventions such as fluid resuscitation, medication adjustment, or nephrology consults. The multi-task framework allows simultaneous prediction of dialysis requirement, providing actionable information for resource allocation and patient management. From a clinical implementation standpoint, the model’s compatibility with both time-series and static data broadens its potential use cases, including resource-limited settings where continuous monitoring may not be available. The attention-weighted output enhances interpretability, which is critical for clinician trust. Integration with electronic health records (EHRs) through real-time data streaming could enable automated alerts for impending AKI. However, before clinical deployment, prospective validation and user-interface adaptation will be essential to ensure seamless integration into existing workflows.

To evaluate the role of symbolic AI components in ChronoNet, we performed an ablation experiment where these modules were removed. The symbolic AI mechanisms in our full model include rule-based filters derived from AKI clinical guidelines, ontology-informed attribute priors, and weak supervision from medical knowledge graphs. As shown in Table 7, removing these symbolic components leads to a noticeable drop in predictive performance across both the MIMIC-III and CKD datasets. In particular, AUC drops by over 2% on both datasets, and F1 score drops by more than 1.5%, indicating the symbolic module enhances generalization and improves precision-recall alignment. These results empirically validate that structured clinical knowledge meaningfully complements the deep learning backbone in real-world AKI prediction tasks.

**TABLE 7:** Effect of symbolic AI integration on ChronoNet performance.

Model	Accuracy	Recall	F1 score	AUC
MIMIC-III ICU Dataset				
ChronoNet (Full)	93.55 ± 0.02	91.67 ± 0.03	92.45 ± 0.01	94.61 ± 0.02
ChronoNet-w/o-SymbolicAI	91.14 ± 0.03	88.23 ± 0.04	89.75 ± 0.02	91.95 ± 0.03
CKD Clinical Dataset				
ChronoNet (Full)	91.67 ± 0.02	89.48 ± 0.03	90.31 ± 0.01	93.10 ± 0.03
ChronoNet-w/o-SymbolicAI	88.02 ± 0.03	84.65 ± 0.03	86.12 ± 0.02	89.27 ± 0.02

To provide transparency regarding class imbalance, Table 8 presents the distribution of AKI-positive *versus* negative cases across the datasets used in this study. The original distributions were heavily skewed, with minority class proportions ranging from 13.5% to 24.3%. To address this, we employed SMOTE to generate synthetic AKI-positive instances, achieving a near-balanced distribution in each dataset. We further applied class-weighted binary cross-entropy and focal loss to ensure the model’s learning remained sensitive to rare but clinically critical events. This combination led to an improvement of 3.1% in AKI recall and 2.7% in F1 score compared to the baseline model trained without imbalance handling. These results confirm that ChronoNet’s training pipeline effectively mitigates bias toward the majority class.

**TABLE 8:** Original and Post-SMOTE class distribution across datasets.

Dataset	AKI positive (before)	AKI positive (after)	Ratio (post)
MIMIC-III ICU	3,812/27,963 (13.6%)	13,187/27,963 (47.1%)	1:1.12
CKD Clinical Dataset	267/1,100 (24.3%)	495/1,100 (45.0%)	1:1.22
General ICU Dataset	2,464/18,204 (13.5%)	8,413/18,204 (46.2%)	1:1.16

To empirically validate the effectiveness of the entropy-based attention regularization introduced in Equation 20, we performed an ablation study by removing this component from the ChronoNet model and retraining it across all benchmark datasets in Table 9. The results reveal a consistent degradation in performance metrics—particularly AUC and F1 score—across both static and dynamic datasets. For example, in the MIMIC-III ICU dataset, the AUC dropped from 94.61% to 91.92%, and the F1 score declined from 92.45% to 89.13%. This performance reduction was most pronounced in cases with irregular or sparse input sequences, which suggests that the entropy regularization plays a critical role in stabilizing the attention distribution. By encouraging a smoother, more diverse allocation of attention weights across time steps, the entropy term reduces the risk of the model overly focusing on a narrow window of temporal data. This is especially important in clinical settings where early indicators of AKI may be distributed across a broader range of time points. The inclusion of this regularization strategy contributes not only to performance gains but also to improved interpretability and reliability of temporal reasoning in high-stakes medical applications.

**TABLE 9:** Ablation study on entropy-based attention regularization across datasets.

Dataset	Setting	AUC (%)	F1 score (%)	Accuracy (%)
MIMIC-III ICU	With Entropy Regularization	94.61	92.45	93.55
	Without Entropy Regularization	91.92	89.13	91.04
Metabolomics	With Entropy Regularization	95.30	93.67	94.38
	Without Entropy Regularization	91.87	89.54	91.93
CKD Dataset	With Entropy Regularization	93.10	90.31	91.67
	Without Entropy Regularization	89.46	86.78	89.52
General ICU	With Entropy Regularization	94.35	92.11	92.15
	Without Entropy Regularization	90.82	88.23	90.47

## 4 Discussion

To provide a broader context for the proposed ChronoNet model, it is essential to compare it with other contemporary architectures in the field of clinical prediction. One notable baseline is RETAIN (Reverse Time Attention Model), which applies a dual-level attention mechanism over RNNs to enable interpretable predictions from sequential EHR data. While RETAIN is notable for its focus on interpretability, it is constrained by its reverse-time dependency and limited flexibility in handling irregular time intervals or dynamic input lengths. In contrast, ChronoNet employs a forward-time LSTM augmented with entropy-regularized temporal attention, allowing it to handle sparse, real-time ICU data more effectively. Another category of interest is Transformer-based models such as Med-BERT or BEHRT, which leverage self-attention for long-range dependency modeling. Although these models perform well with large-scale structured records, they often require extensive pretraining and lack the clinical interpretability necessary for real-time interventions. ChronoNet distinguishes itself by striking a balance between computational tractability and prediction transparency. It incorporates symbolic AI modules, smoothness-aware loss regularization, and adaptive temporal alignment—all of which are designed with clinical workflows in mind. These hybrid strategies enable ChronoNet to generalize across diverse clinical contexts while maintaining interpretability and operational efficiency. Thus, although it shares conceptual elements with existing attention-LSTM or Transformer models, ChronoNet provides a uniquely integrated framework optimized for acute kidney injury prediction under practical constraints.

Although ChronoNet has demonstrated strong predictive performance across multiple datasets, all of these datasets are derived from institutional sources that share similar clinical documentation standards and population structures. As a result, our current evaluation may not fully reflect the challenges associated with deploying AI systems in heterogeneous clinical environments. We recognize this as a limitation that affects the external validity of our findings. Real-world applicability demands that predictive models generalize well across different hospitals, geographical regions, and patient demographics, each of which may exhibit distinct data formats, variable definitions, and clinical protocols. Unfortunately, no external datasets from institutions outside of the current data scope were available during this study for testing such generalizability. To address this gap, future work will focus on conducting external validation through collaborations with other hospitals and health networks. We also recognize that direct data sharing is often restricted due to privacy and regulatory concerns. Therefore, techniques such as federated learning and domain adaptation offer practical avenues for testing and improving cross-site performance without transferring sensitive patient data. These methods will allow the model to learn institution-specific patterns while preserving the shared predictive structure across settings. By explicitly acknowledging and planning for this limitation, we aim to provide a clear and realistic roadmap toward clinical deployment and broader applicability of ChronoNet in diverse real-world environments.

## 5 Conclusion and future work

In this study, we address critical issue of early detection and prediction of Acute Kidney Injury (AKI), a condition that leads to a rapid decline in kidney function. Traditional diagnostic methods, which rely on biomarkers like serum creatinine, often fail to detect AKI at its early stages, thus impeding timely interventions. To address this limitation, we introduce an innovative framework that combines static clinical attributes with temporal dynamics through a deep learning architecture built upon Long Short-Term Memory (LSTM) networks. This architecture is tailored to model the progression of kidney injury over time by leveraging sequential patient data, such as serum creatinine, urine output, and blood pressure measurements. Furthermore, an attention mechanism is incorporated into the LSTM architecture to highlight critical time points essential for predicting AKI. Our experiments reveal that this advanced model outperforms traditional methods in terms of prediction accuracy and early detection, showcasing its potential for clinical application and timely patient intervention. The attention mechanism aids the model in identifying informative intervals, even in shorter sequences, thereby enhancing robustness to sparsity and irregular sampling. These properties make ChronoNet adaptable to real-time applications where the full history may not always be available, reinforcing its clinical applicability.

A fundamental requirement for clinical AI systems is the ability to generalize across diverse patient populations and healthcare environments. While our experiments demonstrate strong performance on multiple large-scale datasets, these datasets are derived from structured and relatively homogeneous sources. Therefore, external validation using completely independent cohorts is essential to confirm model robustness and ensure real-world applicability. Such validation can be carried out by deploying the trained ChronoNet model on datasets from different hospitals or regions, ideally with varying demographics, treatment protocols, and data acquisition systems. However, this process presents several challenges. Data heterogeneity—such as inconsistent variable naming, missing fields, or different measurement units—can complicate preprocessing and alignment. Institutional constraints related to patient privacy and data-sharing agreements may restrict access to necessary validation cohorts. To address these issues, federated learning and domain adaptation techniques offer promising avenues, enabling model refinement without requiring direct data transfer. In future work, we plan to collaborate with external clinical partners to evaluate the model on additional datasets and investigate domain generalization strategies to enhance cross-site transferability.

However, there are two key limitations in this approach that need addressing. First, the model heavily depends on the quality and availability of time-series data, which may not be consistently in all clinical settings. Second, despite its promising results, the model’s generalizability across diverse patient populations and healthcare environments requires further validation. Future research should focus on enhancing the robustness of the model by expanding its training data to include more diverse patient profiles and integrating it with other clinical tools for a comprehensive approach to AKI management.

## Data Availability

The original contributions presented in the study are included in the article/Supplementary Material, further inquiries can be directed to the corresponding author.
